# Coryphoid Palm Leaf Fossils from the Maastrichtian–Danian of Central India with Remarks on Phytogeography of the Coryphoideae (Arecaceae)

**DOI:** 10.1371/journal.pone.0111738

**Published:** 2014-11-13

**Authors:** Rashmi Srivastava, Gaurav Srivastava, David L. Dilcher

**Affiliations:** 1 Cenozoic Palaeoflorist Laboratory, Birbal Sahni Institute of Palaeobotany, 53 University Road, Lucknow- 226 007, Uttar Pradesh, India; 2 Department of Geology, Indiana University, 1001 E. Tenth St. Bloomington- 47405, Indiana, United States of America; Institute of Botany, China

## Abstract

**Premise of research:**

A large number of fossil coryphoid palm wood and fruits have been reported from the Deccan Intertrappean beds of India. We document the oldest well-preserved and very rare costapalmate palm leaves and inflorescence like structures from the same horizon.

**Methodology:**

A number of specimens were collected from Maastrichtian–Danian sediments of the Deccan Intertrappean beds, Ghughua, near Umaria, Dindori District, Madhya Pradesh, India. The specimens are compared with modern and fossil taxa of the family Arecaceae.

**Pivotal results:**

*Sabalites dindoriensis* sp. nov. is described based on fossil leaf specimens including basal to apical parts. These are the oldest coryphoid fossil palm leaves from India as well as, at the time of deposition, from the Gondwana- derived continents.

**Conclusions:**

The fossil record of coryphoid palm leaves presented here and reported from the Eurasian localities suggests that this is the oldest record of coryphoid palm leaves from India and also from the Gondwana- derived continents suggesting that the coryphoid palms were well established and wide spread on both northern and southern hemispheres by the Maastrichtian–Danian. The coryphoid palms probably dispersed into India from Europe via Africa during the latest Cretaceous long before the Indian Plate collided with the Eurasian Plate.

## Introduction

Palms (Arecaceae/Palmae) are considered an important and characteristic component of tropical rainforest ecosystems having a pantropical distribution [1]. The family has been placed within the commelinid clade of the monocotyledons [2], [3], and is composed of five subfamilies: Arecoideae, Calamoideae, Ceroxyloideae, Coryphoideae and Nypoideae [4], [5]. The family comprises 188 genera and about 2600 species [5], [6], [7]. Palm species richness is the highest in tropical Asia (>1200 species) and the higher in the Americas (730 species) than in Africa (only 65 species) [5]. It has been suggested that the low diversity of palms in Africa in contrast to Asia and America is due to Neogene aridification in Africa [8]. However, recent studies suggest *in situ* diversification in other regions like Asia and America etc [9], [10]. In Indian subcontinent, palms consist of 20 genera and 88 species [5] with 24 species belonging to 9 genera endemic [11]. Among the five subfamilies of the Arecaceae, Coryphoideae is sister to a clade comprising Arecoideae and Ceroxyloideae. Asmussen et al. [12] considered Coryphoideae as one of the earliest diverging members of Arecaceae from which both pinnate and palmate leaves may have evolved. However, Baker and Couvreur [9], [10] on the basis of molecular data suggest that the divergence of Coryphoideae occurred at about 87 Ma (95% HPD 86–88) in Laurasia in which *Sabalites carolinensis* Berry described from the late Coniacian–early Santonian (85.8–83.5 Ma) of South Carolina, USA was used as a calibration point [13]. Kvaček and Herman [14] recorded *S. longirachis* Kvaček and Herman from the early Campanian of Austria. A large number of fossil records attributed to Coryphoideae in the form of fruit and wood are also reported from the Deccan Intertrappean sediments. These are: *Hyphaeneocarpon indicum* Bande, Prakash and Ambwani [15], *Palmocarpon coryphoidium* Shete and Kulkarni [16], *Palmocaulon costapalmatum* Kulkarni and Patil [17], *P. hyphaeneoides* Shete and Kulkarni [18], *Palmoxylon coryphoides* Ambwani and Mehrotra [19] and *P. hyphaeneoides* Rao and Shete [20].

**Figure 1:**
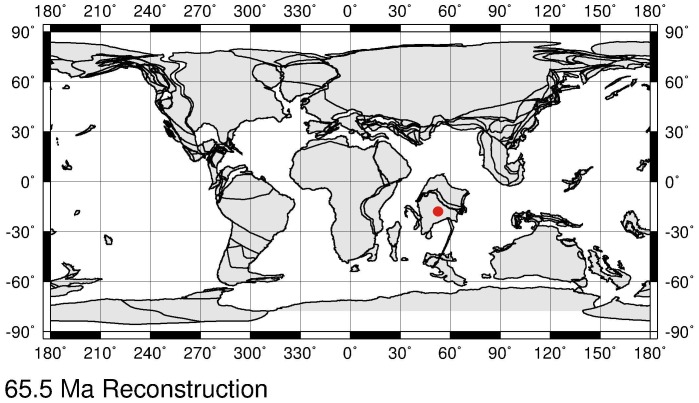
Palaeocontinental map showing the position of India and fossil locality (red dot) at 65.5 Ma [21].

Here we report very rare and well-preserved costapalmate palm leaves under the organ genus *Sabalites* (*S. dindoriensis* sp. nov.) from the Deccan Intertrappean sediments (Maastrichtian–Danian) of Central India. This is the oldest fossil record of costapalmate palm leaves from India and the Gondwana- derived continents. The locality bearing the fossils was situated in a low palaeolatitude ∼18.09° S near the equator (Fig. 1) [21] when the leaves were deposited. Attempts have also been made to discuss the origin and phytogeography of the subfamily Coryphoideae in Indian context.

### The Deccan Traps: a brief review

**Figure 2:**
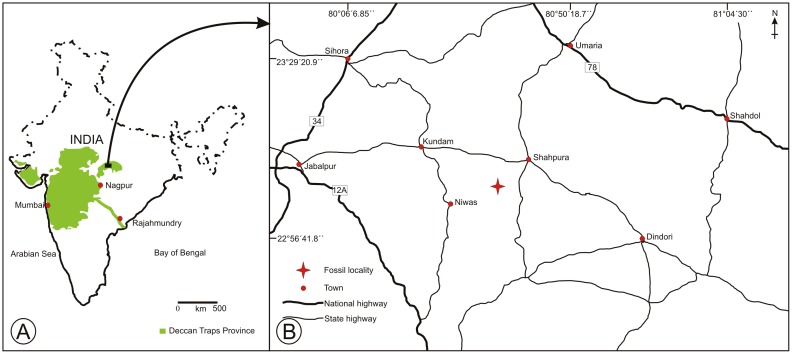
Map of India showing fossil locality. A. Map of India showing extent of Deccan traps. B. High resolution map showing the fossil locality (marked by asterisk) [95].

The Deccan Traps (Continental Flood Basalt) are one of the largest igneous provinces of the world. The area occupied by the Deccan Traps today is about 500,000 sq km in peninsular India which includes Andhra Pradesh, Gujarat, Karnataka, Madhya Pradesh and Maharashtra (Fig. 2A). The original stretch may have been over 1.5 million sq km including sediments found in the Arabian Sea to the west of Mumbai [22]. The outpouring of magma/lava was associated with the northward voyage of the Indian Plate after it was separated from Gondwana during the Early Cretaceous and moved over the Reunion Hot Spot situated east of Madagascar in the Indian Ocean [23], [24]. The extensive volcanic eruptions with associated magma and lava outpouring that formed the Deccan Traps and associated sedimentary beds has been difficult to date and thus is an active topic of discussion among geologists and palaeontologists. Recent studies based on ^40^Ar/^39^Ar dating indicate that the duration of the volcanism extended from 69–61 Ma and the major eruptions took place between 67–65 Ma [25], [26] rather than a short duration of only one million years [27].

The sedimentary sequences between two successive magma/lava flows were deposited in lacustrine, fluviatile and palustrine environments during quiescent (inactive) phases of volcanic activity mainly while the Indian Plate was still an isolated land mass moving toward Asia. These are repeated episodic events resulting in the multiple sequences of fossiliferous beds and basalts. The fossiliferous sediments of the intertrappean beds are exposed mainly in Central India, western India and to the south in parts of Andhra Pradesh and Karnataka, including Rajahmundry (Fig. 2A). The age of the Deccan Intertrappean beds was previously thought to be early Palaeogene due to the abundance of angiospermous remains [28], [29]. However, microfloral studies and faunal assemblages suggest a Maastrichtian age for most of the intertrappean exposures but there are a few Palaeocene indicators [30]–[33]. Currently, based on radiometric dating and planktonic foraminifera, the age of the intertrappean sediments is considered to be upper Maastrichtian–Danian [34]–[37].

Recent studies of the sedimentary sequences associated with the Deccan Traps (both Infratrappean and Intertrappean) have been conducted to resolve their role in mass extinction at the *K*–*Pg* boundary. Cripps et al. [38] working on Mumbai Intertrappeans concluded that volcanic activity had hardly any effect on the floristic elements. However, pollen analysis shows distinct floral changes at different stratigraphic levels [32], [33].

The flora reported from Deccan Intertrappean beds is unique and one of the richest fossil plant assemblages in India. The fossil plant assemblage includes all the plant groups ranging from algae to angiosperms [39]. Most of the fossil flora (mainly angiosperms) from intertrappean beds is reported from Central India (Madhya Pradesh and Maharashtra) with only a few elements of the flora reported from western India [40]–[42]. The majority of the plant macrofossils reported from the Deccan Traps are permineralized woods, fruits with only a few leaf impressions [39], however, microfossils have also been recorded [33].

## Materials and Methods

The fossil palm leaves were collected from Umaria near Ghughua (23° 7' N; 80° 37' E), in the premises of Ghughua Fossil National Park, Dindori District, Madhya Pradesh. The fossil site is situated about 76 km east of Jabalpur and spreads over an area of 27.34 ha in Ghughua and Umaria villages (Fig. 2B). The locality is very rich in permineralized angiospermous woods (both palms and eudicots), but leaves and other plant organs are rarely preserved and thus very rarely found. The studied fossil leaf specimens were first cleaned with a chisel and hammer and then photographed in natural low angled light using a 10 megapixel digital camera (Canon SX110). All the figured fossil specimens (Specimen nos 40073–40077) are housed in the repository of Birbal Sahni Institute of Palaeobotany, Lucknow, India. The fossil leaves were compared with the nearest living relatives in the herbaria of the Central National Herbarium, Howrah, Forest Research Institute, Dehradun, National Botanical Research Institute, Lucknow and the website of Royal Botanic Gardens, Kew. Attempts were made to extract pollen from the floral axis but it could not be recovered. The Director of Birbal Sahni Institute of Palaeobotany, Lucknow has permitted to publish the present work (Ref. No. BSIP/RDCC/Publication no. 22).

Read and Hickey [43] gave five basic characters of palm leaves that can be used alone, or in various combinations to differentiate fossil palm leaves. We followed their classification and placed our specimens in the genus *Sabalites* G. Saporta [44] emended Read and Hickey [43], which they proposed for costapalmate fossil palm leaves.

## Results

**Family.** Arecaceae Schultz Sch.

**Subfamily.** Coryphoideae Burnett

**Genus.** *Sabalites* G. Saporta emended Read and Hickey

**Species.** *Sabalites dindoriensis* R. Srivastava, G. Srivastava and D. L. Dilcher, sp. nov.

**Etymology.** The specific epithet is named after the fossil locality.

**Figure 3:**
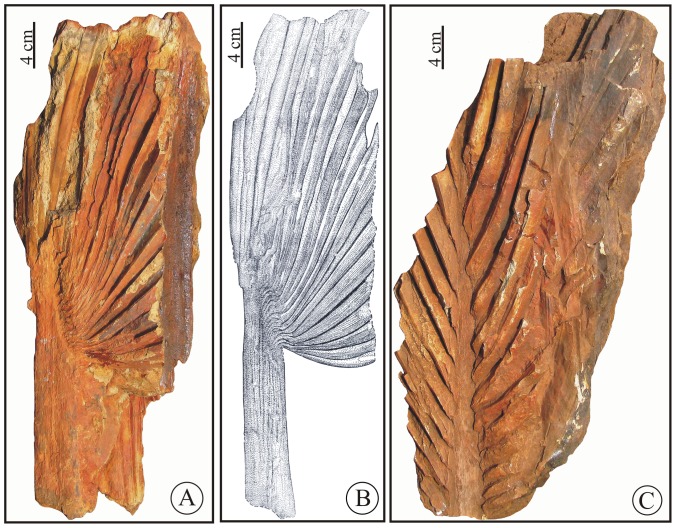
*Sabalites dindoriensis* sp. nov. A. Basal portion of *Sabalites dindoriensis* sp. nov. showing thick costa. B. Drawing of the same fossil. C Middle portion of the fossil leaf showing leaf segments attached to costa.

**Holotype.** BSIP Museum No. 40073, Fig. 3A; designated here.

**Paratypes.** BSIP Museum nos 40074, 40075, 40076, 40077.

**Horizon.** Deccan Intertrappean Beds.

**Type locality.** Umaria near Ghughua Fossil National Park, Dindori District, Madhya Pradesh, India.

**Age.** Maastrichtian–Danian.

### Diagnosis

Leaves costapalmate. Costa/petiole very thick at the basal portion and gradually tapers towards apex, petiole robust, unarmed; a number of longitudinal fibre like structures seen on the petiole/costa. Leaf segments plicate, emerging at an acute angle from costa; fused at emerging point. Mid-veins of each segment thick; two orders of veins on either side of mid-vein, segments near the petiole narrow becoming broader away from the petiole; transverse veins rarely preserved, very fine, perpendicular or obliquely oriented to parallel veins.

**Figure 4:**
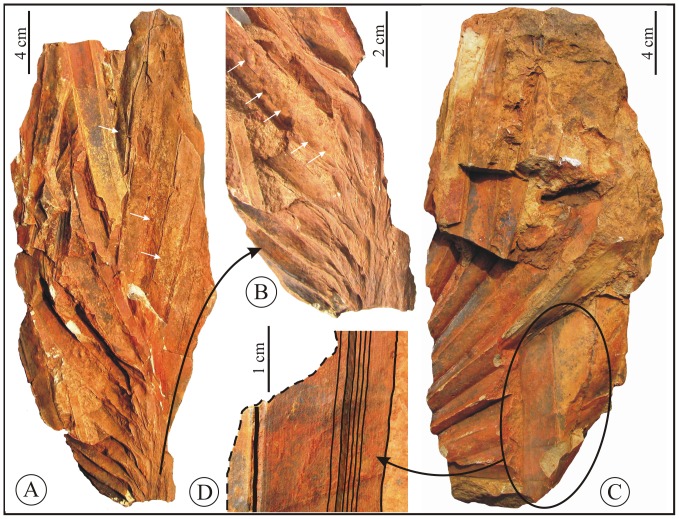
*Sabalites dindoriensis* sp. nov. A. Specimen seems to be of apical portion showing faint impressions of rachilla like structure (white arrows). B. Enlarged portion of the same specimen showing rachilla like structure (white arrows). C. Specimen seems to be of middle portion. D. Enlarged portion showing high order venation.

**Figure 5:**
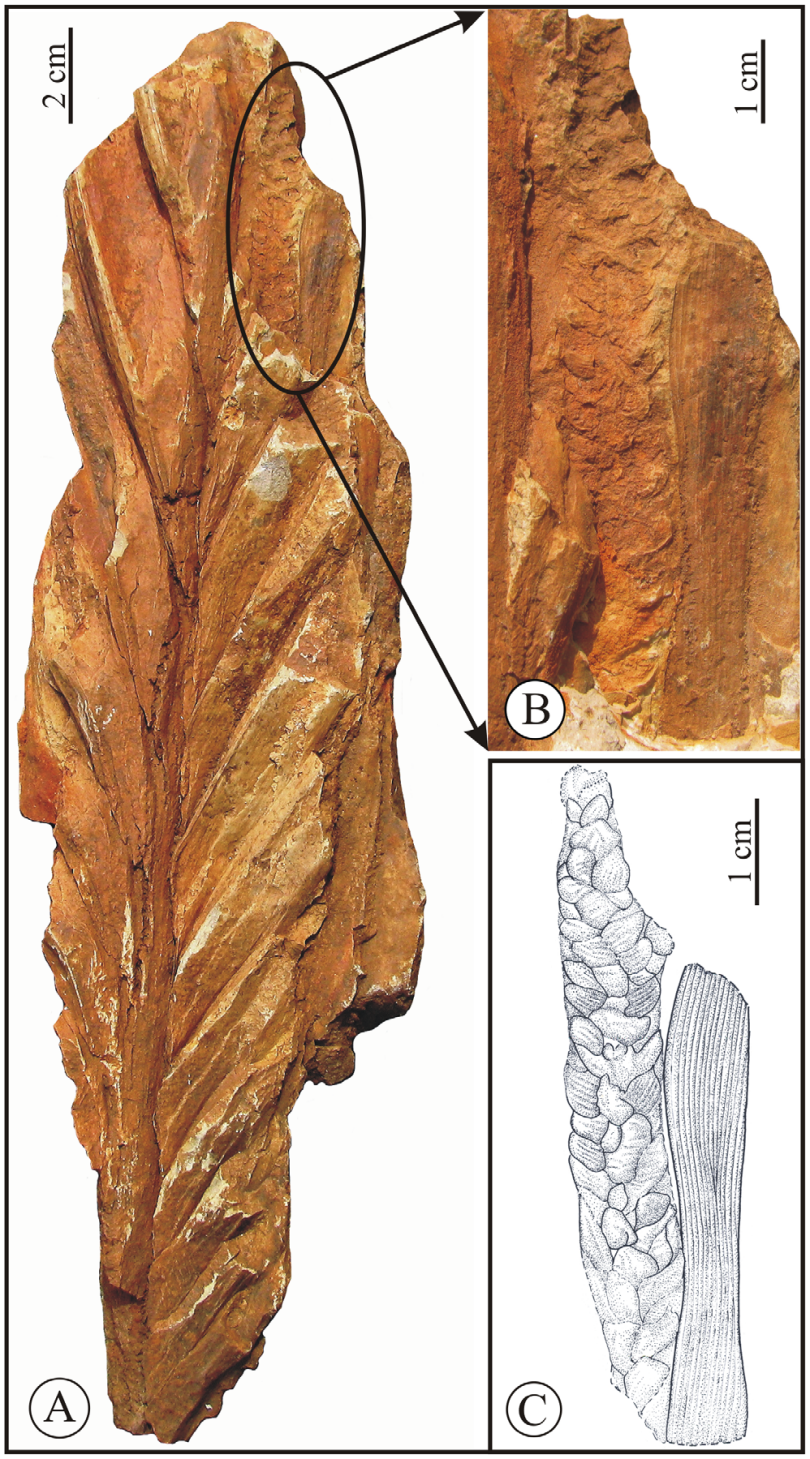
*Sabalites dindoriensis* sp. nov. A. Apical portion of the fossil leaf showing tapering costa with leaf segments having rachilla like structure. B. Enlarged portion of the axis bearing flower showing impression of spirally arranged abscised flowers, bract and spatulate rachillar bract. C. Drawing of the axis bearing flower with bract.

**Description.** The species is described based on the five specimens shown in Figures 3–5. One is the basal part having a thick petiole (Fig. 3A), two specimens (Figs 3C, 4C) are the middle-upper part. Apical portions of two specimens (Figs 4A, 5A) have faint impressions of axis bearing flower. The leaf segments are preserved only near the costa, where they are attached, so the complete size and shape of an entire leaf is uncertain.

*Basal part*: Holotype- BSIP Museum No. 40073, Fig. 3A, 3B.

The preserved length of the specimen is about 45 cm and width about 13.5 cm, petiole with costa is about 26 cm long and 4.2 cm broad at the base that gradually tapers towards the apex and shows attached plicate leaf segments, petiole armature not seen. There are numerous longitudinal fibre like structures present on the petiole and costa. About 15 leaf segments arise from the distal portion of the costa and are crowded together. Leaf segments broaden away from the costa, measuring about 1.0–2.0 cm in width. The mid-veins of leaf segments are about 1–1.5 mm thick and two vein orders present on either side of the mid vein. The higher order venation is not preserved due to the coarse matrix. Transverse veins are rarely preserved but wherever visible, they are very fine and are oriented perpendicularly or obliquely to the parallel veins.

*Middle part*: Paratypes- BSIP Museum nos 40074 and 40075, Figs 3C, 4C.

The specimen in Figure 3C shows the middle portion with a preserved length of about 42.5 cm and a width of 14.5 cm. The costa is about 3.3 cm broad at the base and tapers to 0.7 cm distally. Leaf segments are preserved along both sides of costa but their terminal portions are broken. The segments are inserted on the costa and their maximum length is about 20 cm. The specimen illustrated in Figure 4C contains only plicate leaf segments with a few segments flattened on the rock surface. The detached segments have a maximum width of about 5.4 cm. These leaf segments clearly show a distinct mid-vein with about 16 major parallel veins on either side of mid-vein, each with a minor vein between them (Fig. 4D).

*Apical part*: Paratypes: BSIP Museum nos 40076 and 40077, Figs 4A, 5A.

Specimens (BSIP No. 40076 and 40077) are incomplete and broken. The preserved length is about 30 cm and the width is 7 cm each. The costa tapers gradually from 0.9 cm to 0.3 cm along the apex (Fig. 5A). All the leaf segments are preserved incompletely near the costa; numerous 2° veins run parallel on either side of the midrib.

Also present in this specimen is a poorly preserved impression of a rachilla- like structure (Figs 5A–C) with a preserved length of 9 cm and a width of 1.4 cm. This axis is reminiscent of an inflorescence after the spirally arranged flowers were abscised with striated bracts probably adanate to axis. A spatulate rachillar bract of preserved length 6.6 cm and width 1.4 cm is present adjacent to the floral axis having numerous parallel veins (Figs 5A–C).

**Figure 6:**
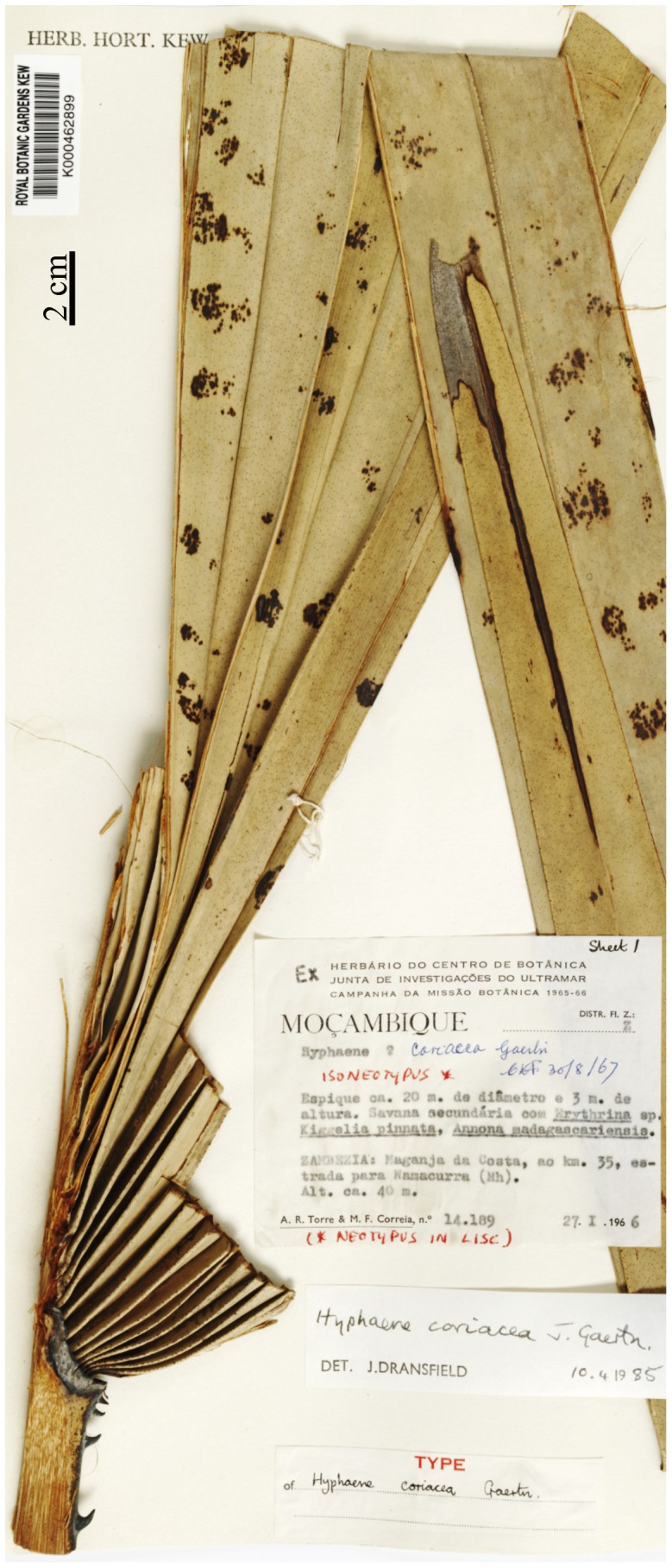
Modern leaf of *Hyphaene coriacea* (modified after http://specimens.kew.org/herbarium/K000462899 [45] **.**

**Affinities.** The diagnostic features of the fossil leaves include: palmate, plicate leaves with long costa (costapalmate) and unarmed petiole. These characters suggest that the fossil leaves have affinities with the subfamily Coryphoideae in the Arecaceae [5]. A number of palm taxa were examined at the Central National Herbarium, Howrah, Forest Research Institute, Dehradun, National Botanical Research Institute, Lucknow and the website of Royal Botanic Gardens, Kew [45] to find species with similar characters. The fossil leaves show resemblance with a number of coryphoid palms with costapalmate leaves in gross morphology such as *Bismarckia nobilis* Hildebr. & Wendl., *Borassus aethiopum* Mart., *B. flabellifer* L., *Corypha taliera* Roxb., *Hyphaene coriacea* Gaertn. (Fig. 6), *H. dichotoma* Furtado, *H. thebaica* Mart., *Livistona australis* Mart., *L. boninensis* Nakai, *L. carinensis* Dransf. and Uhl, *Sabal bermudana* Bailey and *Trachycarpus martianus* H. Wendle. Except *Bismarckia* and *Sabal* all taxa have armed petiole different from our fossil. The leaves of *Trachycarpus* H. Wendl. are non-costate which differentiates it from the present fossil. The inflorescence like structure of the fossil shows a close resemblance with the *Hyphaene* Gaertn. by having characteristic shape, striated bracteoles and spatulate large bract associated with floral axis which also gets support from the previous fossil records of *Hyphaene* from the same horizon [15], [18], [20]. However, due to the lack of spines on the petiole of fossil it cannot be assigned exactly to the modern taxa. As Read and Hickey [43] stated that “Since it is very difficult to identify specimens of modern palms accurately from their leaves alone, no attempt should be made to place fossil palm fragments in genera of modern palms unless unquestionably identifiable with them”. Under these circumstances the fossils are placed in the organ genus *Sabalites* G. Saporta [44] proposed for costapalmate fossil leaves.

A number of palm leaves have been described from Upper Cretaceous–Neogene deposits of India under various fossil taxa (table 1) [46]–[71]. The species attributed to *Amesoneuron* (Goeppert) Read and Hickey [47]–[53] cannot be compared with the leaves under consideration as they are isolated fragments of lamina with parallel veins but the fragments are not attached to main rachis making it impossible to determine whether they belong to pinnate or palmate leaves. *Malpophyllum dakshinens* [58] is based on anatomical features and *Malpophyllum* sp. [58] is based on a very fragmentary specimen in which costa and other characteristic features are not preserved.

**Table 1:** Fossil palm leaves from Upper Cretaceous–Neogene sediments of India.

Fossil species/References	Locality/Horizon	Age
Fossil palm leaf and stem [46]	Polgaon, DIB Nagpur	Maastrichtian
*Amesoneuron borassoides* Bonde [47]	Chhindwara, DIB	Maastrichtian–Danian
*A. deccanensis* Guleria and Mehrotra [48], [50]	Seoni and Dindori, DIB; East Garo Hills; Tura Fm.	Maastrichtian–Danian;Upper Palaeocene
*A. ladakhensis* Mehrotra et al. [49]	Hemis Conglomerate Horizon, Ladakh	Late Eocene–Oligocene
*A. lakhanpalii* Mehrotra [50]	East Garo Hills; Tura Fm.	Upper Palaeocene
*A. manipurensis* Guleria et al. [51]	Imphal	Late Eocene
*sahnii* Guleria et al. [52]	Solan; Kasauli Fm.	Lower Miocene
*A. siwalicus* Prasad [53]	Jawalamukhi, Siwalik	Middle Miocene
Borassiod palm leaf [54]	Chhindwara, DIB	Maastrichtian–Danian
Palm leaves [55]	East Godavari, east of Rajahmundry	Late Tertiary
*Livistona wadiai* Lakhanpal et al. [56]	Hemis Conglomerate Horizon, Ladakh	Late Eocene–Oligocene
cf. *Iguanura wallichiana* Srivastava et al. [57]	Tinsukia, Makum; Tikak Parbat Fm.	Late Oligocene
*Malpophyllum dakshinens* Kumaran [58]	Chhindwara, DIB	Maastrichtian–Danian
*Malpophyllum* sp. [58]	Solan; Kasauli Fm.	Lower Miocene
*Nypa fruticans*Wurmb [59]	Tinsukia, Makum; Tikak Parbat Fm.	Late Oligocene
*Palmacites* sp. [50]	Barail; Tirap	Oligocene
*Palmacites* sp. [60]	Kangra, Ranital; Lower Siwalik	Middle Miocene
*P. khariensis* Lakhanpal and Guleria [61]	Kutch, Khari Series	Miocene
*P. makumensis* Srivastava et al. [57]	Tinsukia, Makum; Tikak Parbat Fm.	Late Oligocene
*P. tsokarensis* Paul et al. [62]	Hemis Conglomerate Horizon, Ladakh	Late Eocene–Oligocene
*Phoenicites* sp. [63], [64]	Tura Fm., Garo Hills Laisong Fm.	Eocene Late Eocene–Oligocene
*P. indica* Guleria et al. [51]	Imphal	Late Eocene
*P. siwalikensis* Bonde [65]	Darjeeling; Middle Siwalik	Miocene
*P. lakhanpalii* Guleria and Mehrotra [48]	Seoni; DIB	Maastrichtian–Danian
*Sabalites* sp. [66], [67]	Near Chakoti river, Jhelum, Kashmir;	Miocene
	Solan, Kasauli Fm.	Lower Miocene
*S. microphylla* Sahni [66]	Solan, Kasauli Fm.	Lower Miocene
*Sabalophyllum livistonoides* Bonde [68]	Nawargaon, DIB	Maastrichtian–Danian
*Trachycarpus ladakhensis* Lakhanpal et al. [69], [70]	Ladakh, Liyan Fm.; Lower Siwalik	Miocene; Early Miocene;
*Zalaccites jaintiensis* Burman and Daura [71]	Khasi and Jaintia Hills	Palaeocene

DIB: Deccan Intertrappean Beds; Fm.: Formation.

*Phoenicites* Brongniart species (*P. lakhanpalii* Guleria and Mehrotra [48]; *P. indica* Guleria et al. [51]; *Phoenicites* sp. [63], [64]; *P. siwalikensis* Bonde [65]), palm leaf [55], *Sabalites* sp. [66], [67], *Zalaccites jaintiensis* Barman and Duara [71] and the leaf of cf. *Iguanura wallichiana* Srivastava, Mehrotra and Bauer [57] are pinnate leaves and therefore differ from *Sabalites dindoriensis*.

Palmate leaf fossil taxa reported from Indian Upper Cretaceous–Neogene sediments includes *Palmacites*, a taxon for leaves lacking primary costa. Costapalmate forms referred to *Sabalites* and *Sabalophyllum* and two specimens placed in the modern genera, *Livistona* and *Trachycarpus*. The *Palmacites* species (*Palmacites* sp. [50], *P. makumensis* Srivastava, Mehrotra and Bauer [57], *P. khariensis* Lakhanpal and Guleria [61], *P. tsokarensis* Paul et al. [62]) lack a costa and therefore differ from *Sabalites dindoriensis*. *Sabalophyllum livistonoides* Bonde [68] is based on a petrified leaf with anatomical features and therefore it could not be compared with *S. dindoriensis*. *Sabalites microphylla* Sahni [66] and *Sabalites* sp. [67] are fragmentary palmate leaves not attached to petiole, so it is not clear whether they are costapalmate. *Trachycarpus ladakhensis* Lakhanpal et al. [69], [70] lacks primary costa. *Livistona wadiai* Lakhanpal et al. [56] is the only costapalmate leaf reported from late Eocene–Oligocene sediments of Ladakh Himalaya, it is a smaller leaf with thin costa and narrower leaf segments.

In a detailed work on coryphoid palms from the Eocene of Texas, Daghlian [72] reported a number of fossil palm leaves under various fossil taxa, namely, *Costapalma, Palmacites, Palustrapalma, Sabal* and *Sabalites* based on cuticular features. But none of them show morphological resemblance with the leaves of *Sabalites dindoriensis*. Recently, Zhou et al. [73] reported many coryphoid palm leaves from the Eocene of southern China. But all the specimens are much smaller in size, with intact cuticular structures and short costa. Besides, the fossil records of palms are abundant worldwide and it is not possible to explore all of them here. Therefore, we compare *Sabalites dindoriensis* with those fossil costapalmate leaves that are most similar. *Sabal chinensis* (Endo) Huzioka and Takahashi [74] from the Eocene of Northeast China, Japan and Russia is similar to the basal part of one specimen (Fig. 3A, BSIP Museum no. 40073) in having a thick stout unarmed petiole extending into the lamina (costapalmate) and leaf segments fused at their emerging point. It differs from the present species by having a shorter costa with narrower segments. The specimens of the middle and apical portions of *Sabilites dindoriensis* (Figs 3C, 4A–D and 5A BSIP Museum nos 40074–40077) show a close resemblance to *S. longirachis* [14] reported from the early Campanian of Austria and Maastrichtian of the Pyrénées [75] in having a thick long costa and segments with a similar angle of attachment. However, this species is based on cuticular features and fine venation, none of which are preserved in *S. dindoriensis* leaves. Therefore, in the absence of any similar leaves we propose the new species, *Sabalites dindoriensis* R. Srivastava, G. Srivastava and D. L. Dilcher sp. nov.

## Discussion

### Modern distribution of palms and their ecology

**Figure 7:**
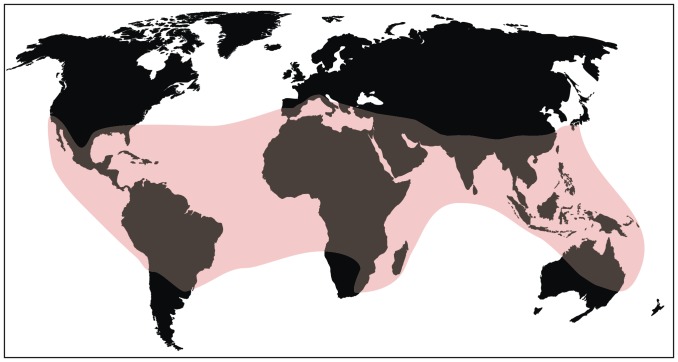
World map showing modern distribution of Coryphoideae [5].

Palms are largely distributed and diversified in tropical areas [5], [76] with 90% of the species diversity restricted to tropical rainforest [1]. They are much less prominent and diverse in temperate regions [5], [77], [78], thus showing very restricted frost tolerance [77]. The low frost tolerance of palms is considered to be an evolutionarily conserved trait. Their architecture and more notably a crown composed of large evergreen leaves [79], which has limited frost resistance [80] and unique stem physiology doesn't allow dormancy [81]. Palms also exhibit a strong latitudinal diversity gradient [82] and need water accessibility for their survival [5], [83]. Palms grow mainly under the top canopy of tropical rain forests along low hills and streams in warm and humid conditions, while a few grow in open areas. They are also dominant in coastal swamps and mangrove forests [8], [84]. Studies of new world palms indicate that solar radiation as related to absolute latitude and water is the main factor that determines the richness of palms species [85]. However, the subfamily Coryphoideae is distributed in a wider range of habitats such as pantropical to warm temperate areas of the world (Fig. 7). It is also found in climatic extremes such as cold and arid regions [5].

### Origin and possible migratory path of the Coryphoideae from the Northern Hemisphere to Indian subcontinent

**Figure 8:**
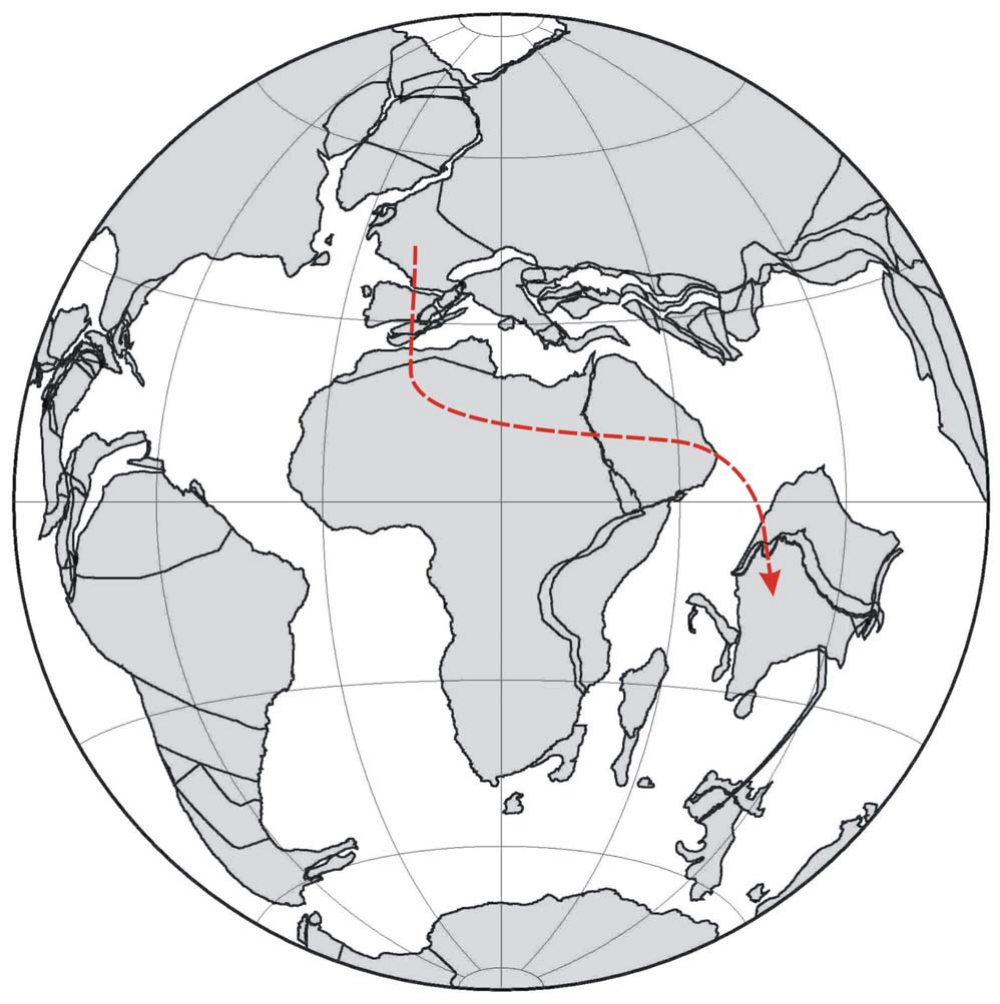
Palaeogeographic map at 65.5 Ma [21] showing possible dispersal path of Coryphoideae from Europe to India via Africa (red broken line).

Phylogenetic and molecular clock studies indicate that the palms originated and diversified in Laurasia around 100 Ma [1], [10] while the coryphoids diverged at about 87 Ma (95% HPD 86–88) which was constrained by the calibration point from a *Sabalites* fossil [9], [10] and diversified during the Late Cretaceous to Cenozoic in boreotropical regions [5], [86]. The subfamily Coryphoideae includes four major clades including (1) New world thatch palm clade consisting of tribes Sabaleae and Cryosophileae; (2) Syncarpous clade consisting of tribes Chuniophoeniceae, Caryoteae, Coryphae and Borasseae; (3) tribe Phoeniceae and (4) tribe Trachycarpeae [5], [87]. Based on molecular phylogenetic analysis, Baker and Couvreur [9] suggested that the New world thatch palm clade diverged at 55 Ma (95% HPD 39–72) in North America. The Syncarpous clade diverged in Eurasia at 66 Ma (95% HPD 51–80). The tribe Phoeniaceae diverged from Trachycarpeae around 49 Ma (95% HPD 33–65) in Eurasia. Out of the four aforesaid clades, the syncarpous clade is the earliest diverging clade (66 Ma) that also corresponds to the age of Deccan Intertrappean beds to which our fossils belong. In syncarpous clade, the Caryoteae can be differentiated from the present fossil by having pinnate or bipinnate leaves while amongst Chuniophoeniceae, Coryphae and Borasseae the fossil probably shows near resemblance with floral axis of *Hyphaene* (Borasseae) by having the characteristic shape and striate bractioles which also corroborate with the previous fossil records of *Hyphaene* from the same horizon [15], [18], [20]. In the subsequent study Baker and Couvreur [10] suggested that only one dispersal event occurred from Indian Ocean into India (including Sri Lanka) during the Miocene but the palm fossils reported from the Maastrichtian–Danian sediments of Deccan Intertrappean beds [39] opens a new dispersal route (Fig. 8).

The oldest fossil records of Coryphoideae are reported from the Northern Hemisphere, such as: *Sabalites carolinensis* [13] from late Coniacian–early Santonian of South Carolina and *Sabal bigbendense* Manchester, Lehman and Wheeler [88] from Maastrichtian of Texas, USA. *Sabalites longirhachis* [14] were reported from the lower Campanian of Austria and from the Maastrichtian of the Pyrénées [75].

The fossil records show that the continent of Africa and India possessed much richer palm flora in the past than at present. In Africa, there are definite evidences of palm pollen from the Campanian (83.5–70.6 Ma) and they became much abundant and more diverse during the Maastrichtian (70–65.5 Ma). This period is referred as ‘Late Cretaceous Palm Province' [8], [89]. The subfamilies such as Nypoideae and Calamoideae have been recorded from the Maastrichtian [90], [91], the Coryphoideae in the form of seed has been recorded from the Danian (65.5–61.7 Ma) sediments of Egypt [92]. A large number of fossils attributed to Coryphoideae have been reported from the Deccan Intertrappean sediments such as: *Hyphaeneocarpon indicum* [15], *Palmocarpon coryphoidium* [16], *Palmocaulon costapalmatum* [17], *P. hyphaeneoides* [18], *Palmoxylon coryphoides* [19] and *P. hyphaeneoides* [20]. It is interesting to note that several of the *Palmoxylon* species reported from the Upper Cretaceous sediments of the Indian subcontinent [66] have also been reported from the late Eocene to early Miocene sediments of Egypt [93].

All the above fossil records and the similarity in coryphoid fossil palm records between India and Africa suggest that the coryphoid palms probably dispersed into India from Europe via Africa. During the Early Cretaceous the Indian plate was separated from the other Gondwana continents and moved northward. It collided with the Kohistan-Ladakh arc at ∼85 Ma (an island like structure) re-establishing the land connection between India and Africa ∼70 Ma [24]. This facilitated the interchange of various plants and also several Maastrichtian dinosaurs [24]. During the Campanian–Maastrichtian, Africa was also connected with Europe by land [94], which most likely facilitated the entry of coryphoid palms from Europe to Africa. Thus, the migration model of coryphoid palms we propose (Fig. 8) fits well with the plate tectonic models. However, with the aforesaid model, long distance oceanic dispersal cannot be ruled out [9], [10]. In future better preserved palm fossils assignable to modern genera are needed to further strengthen the proposed model.
